# The impact of mobile phone based messages on maternal and child healthcare behaviour: a retrospective cross-sectional survey in Bangladesh

**DOI:** 10.1186/s12913-017-2361-6

**Published:** 2017-06-24

**Authors:** Mafruha Alam, Catherine D’Este, Cathy Banwell, Kamalini Lokuge

**Affiliations:** National Centre for Epidemiology and Population Health, Research School of Population Health, The Australian National University, Canberra, Australia

**Keywords:** mhealth, Delayed bathing, Postnatal care visits, Breastfeeding, Bangladesh

## Abstract

**Background:**

Mobile phones are gradually becoming an integral part of healthcare services worldwide. We assessed the association between Aponjon mobile phone based messaging services and practices regarding childbirth and care of mother and neonates in selected areas in Bangladesh.

**Methods:**

In early 2014, 476 subscriber mothers whose last born child’s age was between 3 and 18 months, were recruited to the study by Dnet from selected areas of Bangladesh. One group of mothers received the early warning messages from Aponjon during pregnancy (exposed; *n* = 210) while the other group of new mothers did not receive the messages during pregnancy as they had enrolled in the service after childbirth (non-exposed; *n* = 266). We undertook regression analyses to investigate the relationship between timing of exposure to Aponjon messages and socio-economic factors and outcomes of safe delivery, immediate breastfeeding post birth, delayed bathing of the neonate, and number of postnatal care (PNC) visits.

**Results:**

Women reported delivering babies at home without a skilled birth attendant (SBA) (*n* = 58, 12%), at home with SBA (*n* = 111, 23%) and at health facilities (*n* = 307, 65%). Most (*n* = 443, 93%) women breastfed babies immediately post birth. Babies were bathed after 72 h (*n* = 294, 62%), between 48 and 72 (*n* = 100, 21%) and between 0 and 47 (*n* = 80, 17%) hours after birth. PNC frequencies were reported as none (*n* = 273, 57%), 1 (*n* = 79, 17%), 2 (*n* = 54, 11%), 3 (*n* = 34, 7%) and 4 (*n* = 36, 8%). There was no significant association between exposure to Aponjon messages during pregnancy and presence of a SBA at birth, breastfeeding practices, and postnatal care visits, although delayed bathing up to 48 h was significant at the 10% but not 5% level (RRR 1.7; 95% CI 0.93–3.0; *p* = 0.083). Women with higher education, from higher income, older in age, with birth order 1 or 2 were more likely to birth at health facilities. Facility based delivery was an independent factor for delayed bathing and having postnatal care visits.

**Conclusions:**

Low cost mobile phone messages may have the potential to positively influence maternal and child healthcare behaviours, such as delayed timing of first bath, in resource-poor settings. Further studies are needed, with adequate sample size to detect significant change.

**Electronic supplementary material:**

The online version of this article (doi:10.1186/s12913-017-2361-6) contains supplementary material, which is available to authorized users.

## Background

Neonatal mortality remains unacceptably high in resource limited countries such as Bangladesh where the rate currently stands at 28 per 1000; 61% of under-5 child mortality [1]. Bangladesh aims to reduce neonatal mortality to 21 per 1000 live births by 2016 although it is still unclear how this might be most effectively achieved [2].

One avenue for reducing neonatal mortality is to improve compliance with the World Health Organization﻿'s﻿ (WHO) recommendations concerning delivery and neonatal care. Currently, in Bangladesh, less than half (42%) of deliveries are assisted by a medically trained personnel and slightly more than a third of mothers (39%) and new-born babies (36%) receive timely postnatal care (PNC) from a trained provider [1, 3, 4]. In contravention of WHO guidelines, 32% of babies in Bangladesh are bathed within the first 6 h of life putting them at risk of hypothermia and 49% of new-born babies do not receive breast milk in their first hour of life increasing their risk of infection [1, 5–8].

Mobile phones have recently been adopted to disseminate health information and interventions (mHealth) in low and middle income countries [9]. In Bangladesh there has been a rapid proliferation of mobile phone usage throughout the country since 1998. By 2012, there were more than 90 million mobile phone subscribers, creating an opportunity for personalized access to health information for all [10]. The health infrastructure of the country has undergone major reforms and the Ministry of Health and Family Welfare (MOHFW) now provides a platform for public-private mHealth initiatives in Bangladesh [11].

One such initiative is the Aponjon service which provides weekly voice or text messages primarily to pregnant women and mothers with new born babies to improve maternal and child healthcare outcomes [12]. The service was officially launched in 2012 after a year-long pilot study and is available through all telecommunication operators in the country to a subscriber base of more than a million people. The United States Agency for International Development (USAID) provided a seed grant and the project was implemented by Dnet, a social enterprise. Aponjon receives in-kind grants from corporate companies and collaborates with local Non-Government Organizations (NGOs) to promote the service across the country [13].

Generally, there is a lack of evidence on the effectiveness of scalable mHealth services [14]. There are few peer-reviewed studies on implementation and evaluation of mHealth programmes in low and middle-income countries around the world [15]. Although randomized trials elsewhere show that mobile phone based reminders increase maternal attendance at hospitals, skilled birth attendance in delivery, exclusive breastfeeding, immunization, adherence with family planning methods and other best practices, ambiguous descriptions of interventions and their mechanisms of impact present difficulties for interpretation and replication [16–21]. Most studies of mHealth in low and middle income countries lack rigorous methodologies and are unable to draw robust conclusions about impacts on patient health outcomes [21]. Aponjon is the first national scalable mHealth service in Bangladesh, however, currently little is known about how mHealth services like this will be integrated into the existing health system [22].

The aim of this cross-sectional study of Aponjon subscribers collected in 2014 by Dnet is to evaluate the effectiveness of a mobile phone based intervention in Bangladesh designed to improve compliance with WHO guidelines related to delivery and neonatal care: assistance of a skilled birth attendant during delivery, breastfeeding within an hour after birth, timing of the baby’s first bath and the number of routine postnatal care visits within the first 42 days. This study also explores other factors that impact on maternal and new-born care practices.

## Methods

### Aponjon service

The Aponjon service offers information on pregnancy, delivery, essential new born care and nutrition for pregnant women and new mothers via mobile phone voice or text messages. A woman can enrol in the service any time during her pregnancy and continue to receive the phone messages until the baby turns 1 year of age. She can also enrol in the service after her baby is born and receive messages appropriate for her baby’s stage of growth until the baby turns 1 year of age. Interested families can dial up a numerical short code which was widely disseminated through the media to enrol in the service. Health workers of partner NGOs also recruited women during monthly door-to-door routine antenatal care visits. The service is provided in Bangla, in either recorded voice messages or in text format, subject to individual choice. Voice messages are 1 min long while text messages are limited to 161 characters and only appropriate for people who can read transliterated Bangla. A woman can also include her husband or other nominated family member in the service. Throughout the service period, a woman receives 2 messages a week, while her nominated family member will receive 1 message a week. Each message cost only BDT 2.3 (0.02 USD) to keep the service within reach of general population.

### Survey

Data were obtained from a survey conducted by Dnet as a part of routine operations research to monitor the Aponjon service milestones. The survey was conducted between February–April 2014 in five (Bagerhat, Bogra, Chittagong, Laxmipur and Patuakhali) of the 64 districts in Bangladesh. The survey districts were purposively selected to reflect the remoteness, diversity, geographical dispersion and maximum acquisition of subscribers. From within these districts, Dnet recruited participants from their database of subscribers which contained women’s cell phone numbers, location and service history. As a result 2433 new mothers who had been exposed to the service for at least for 3 months during pregnancy or after childbirth, who had a live birth in the last delivery and whose last born child’s age was between 3 and 18 months were eligible. Due to the availability of health workers to undertake the survey, a maximum of 966 women could potentially be contacted. Women were systematically sampled district by district, and phoned to seek verbal consent and to arrange a date and time to be interviewed, until the required number had been recruited. Face-to-face interviews were conducted in participants’ homes by two researchers, including one woman, to build rapport with respondents. Information on the survey, including protection of confidentiality, was read aloud to potential respondents and written consent was taken before each interview. The interviews were conducted in Bangla using a semi-structured questionnaire and lasted about an hour. Respondents were excluded from the survey if they could not be contacted by phone for an appointment, were not at home when the interviewers visited to conduct the interview, or they did not want to participate. In most of the cases women who were not at home or were visiting relatives outside the survey areas for an indefinite time were not followed up for interviews.

The survey asked about subscribers’ access to messages, the comprehension and generalizability of messages, the influence of the service on subscribers’ knowledge of maternal and child health and user satisfaction with the service (Additional file 1). This study received approval to analyse the survey data collected by Dnet in 2014 from Dnet and from the Humanities & Social Sciences Delegated Ethics Review Committee (DERC) of Australian National University.

### Measures

One group of new mothers was enrolled and exposed to the messages during pregnancy, and the other group was enrolled in the service after delivery and were not exposed to the messages during pregnancy. These two groups were defined as “exposed” and “not-exposed” respectively, based on the timing of their inclusion in the service as indicated in the service database. The ratio of exposed to unexposed women varied across districts.

**Fig. 1:**
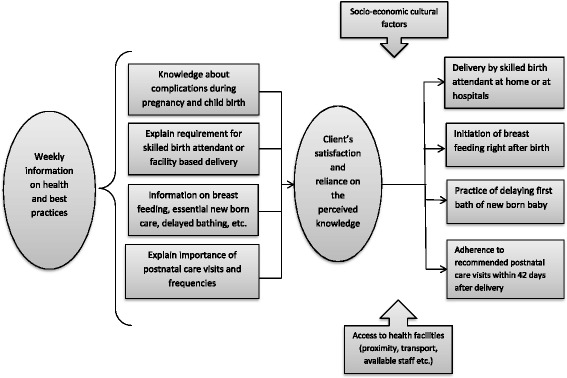
Conceptual Framework

Aponjon provides repeat early warning messages to pregnant women based on current WHO guidelines which: encourage women to plan their delivery with a skilled birth attendant (SBA) at home or at a health facility, initiate colostrum feeding within an hour of birth, delay bathing of the new-born baby up to 3 days, and ensure 4 postnatal care visits of the mother and new-born within 42 days of delivery. We propose that subscribers who received the early warning messages during pregnancy would be more likely to undertake these health practices compared to women who did not receive messages until after the birth of the baby (Fig. 1).

The outcomes of interest were based on the overall aims of the Aponjon program and the messages delivered to Aponjon subscribers. The first outcome “assistance during delivery” derived from two survey questions. Women were asked “Where was your baby born?” and “Who assisted the delivery (in case of home delivery)?”. These responses were then grouped into: “unskilled birth attendant at home”; “skilled birth attendant at home” and “trained personnel at health facility”.

The second outcome “breast feeding immediately post birth” was generated as a binary variable from the question “What did you feed your baby right after birth?”. The response option “fed breast milk” was coded as “Yes” and “fed honey, sweetened water or mustard oil” was coded as “No”.

From the question “When did you first bathe your baby after birth?” emerged the third outcome variable “timing of first bath”. Aponjon, following the national guidelines, recommends that the first bath is given at least 72 h after delivery. Because the risk of hypothermia is higher with earlier timing of first bath, the outcome variable has been categorized into three groups: 0–47 h, 48–72 h and 72 + hours. The forth outcome variable “frequency of postnatal care visits” (by a medically trained provider) within 42 days of delivery is a count variable, with responses 0, 1, 2, 3, 4.

Other potential covariates were based on factors demonstrated to be associated with maternal and neonatal outcomes in previous research, or hypothesised to be important in this study [23]. They were: age (<25 years and 25 years or more), women’s education (no or primary education, junior secondary education and secondary education), family income (less than 10,000 BDT and BDT 10,000 or more), birth order (1, 2 and 3 or more), district and place of residence (urban or rural). We considered facility based delivery (at health facilities or at home), a part of the first outcome, as a covariate for other outcomes.

### Statistical methods

The sample characteristics are described using numbers and percentages. Bivariate analysis was performed to compare characteristics between exposure groups and to examine associations between explanatory and outcome variables using Chi-squared tests.

We undertook multiple regression analyses to investigate the relationship between “exposure to early warning messages” and the outcomes of interest, adjusted for women’s age, education, family income, place of residence, birth order and district (to account for the sampling strategy) using a model appropriate for the distribution of each outcome: multinomial regression analysis for “assistance during delivery” and “timing of first bath”, logistic regression analysis for “breast feeding immediately post birth”, and negative binomial regression for “frequency of postnatal care visits”. “Facility based delivery” was included in all of the above models except in the model for “assistance during delivery”. The results are expressed as Relative Risk Ratios (RRR) for multinomial, Odds Ratio (OR) for binary logistic and Incidence Rate Ratios (IRR) for negative binomial regression models, with 95% confidence intervals.

Dnet estimated a sample size of approximately 400 was required, with an anticipated ratio of exposed to unexposed of 1:2, to detect a difference in proportions with outcomes of interest between exposed and unexposed of 15%, with 80% power and a 5% significance level. Assuming a consent rate of 60%, it was expected that 660 Dnet subscribers would need to be contacted for the study.

## Results

Of 660 Dnet subscribers contacted, 476 (72%) respondents were interviewed (exposed =210, non-exposed =266). The socio-demographic characteristics of the 476 survey participants are shown in Table 1. The respondents were mostly recipients of voice (*n* = 470, 99%) messages rather than text (*n* = 6, 1%) messages. There is no significant difference in age and socio-economic background of the two groups except there is significant difference in districts and age of last born children. In district D there are more respondents in the exposed group and in district E more respondents are in the non-exposed group. Significantly more children were less than 6 months age (51%) in the exposed group while exposed group had more babies belonging to the age group 6–12 months (44%).

**Table 1:** Socio-demographic characteristics of respondents

Characteristic	Exposed (*n* = 210)	Non-exposed (*n* = 266)	Chi-squared test
Age of respondent women			
< 25 years	129 (61%)	148 (56%)	χ2 = 1.617; df = 1; *p* = 0.204
≥ 25 years	81 (39%)	118 (44%)	
Family monthly income			
< 10,000 BDT	107 (51%)	116 (44%)	χ2 = 2.541; df = 1; *p* = 0.111
≥ 10,000 BDT	103 (49%)	150 (56%)	
Women’s education			
None or primary education	86 (41%)	100 (37%)	χ2 = 0.576; df = 2; *p* = 0.757
Junior secondary school	65 (31%)	87 (33%)	
Secondary school or higher	59 (28%)	79 (30%)	
Access to sanitary latrine			
Yes	194 (92%)	244 (92%)	χ2 = 0.068; df = 1; *p* = 0.795
No	16 (8%)	16 (8%)	
Access to potable drinking water			
Yes	209 (99%)	263 (99%)	χ2 = 0.598; df = 1; *p* = 0.439
No	1 (1%)	3 (1%)	
Residence			
Urban	134 (64%)	127 (48%)	χ2 = 12.229; df = 1; *p* < 0.001
Rural	76 (36%)	139 (52%)	
^a^ Districts			
District A	33 (16%)	28 (11%)	χ2 = 36.524; df = 4; *p* < 0.001
District B	44 (21%)	72 (27%)	
District C	62 (29%)	53 (20%)	
District D	40 (19%)	22 (8%)	
District E	31 (15%)	91 (34%)	
Number of living children			
1	91 (43%)	112 (42%)	χ2 = 1.452; df = 2; *p* = 0.484
2	73 (35%)	105 (40%)	
≥ 3	46 (22%)	49 (18%)	
Age of last born child (living)			
< 6 months	107 (51%)	52 (20%)	χ2 = 66.775; df = 2; *p* < 0.001
6–12 months	84 (40%)	125 (47%)	
13–18 months	19 (9%)	89 (33%)	
Type of messages received			
Voice	207 (99%)	263 (99%)	χ2 = 0.085; df = 1; *p* = 0.770
Text	3 (1%)	3 (1%)	
Woman’s access to phone			
Shares phone	92 (44%)	119 (45%)	χ2 = 0.001; df = 1; *p* = 0.969
Never shares phone	118 (56%)	147 (55%)	

^a^Districts name have been labelled to maintain anonymity

Approximately two-thirds of participants were assisted during delivery by “trained personnel at a health facility” (*n* = 307, 65%) while 111 (23%) women reported the presence of a skilled birth attendant and the rest (*n* = 58, 12%) were assisted by untrained relatives or local traditional birth attendants at a home delivery (Table 2). Exposure to messages during pregnancy was not statistically significantly associated with the presence of an unskilled birth attendant at a home delivery (RRR: 1.6; 95% CI 0.84–2.9; *p* = 0.156) or with a skilled birth attendant at home (RRR: 1.2; 95% CI 0.71–1.9; *p* = 0.514) relative to a health facility delivery. Women’s education and family income were statistically significantly associated with the assistance of an unskilled birth attendant during delivery. Women who had no education or primary education only (RRR: 12.5; 95% CI 3.5–45.0; *p* < 0.001) or those with junior secondary education (RRR: 6.4; 95% CI 1.8–22.9; *p* = 0.005) were more likely than women who completed secondary education to deliver at home by an unskilled birth attendant rather than at a health facility. Similarly, women with a lower family income (RRR: 2.6, 95% CI 1.3–5.1; *p* = 0.005) were more likely to give birth at home with an unskilled birth attendant than at health facilities. Women with none or primary education (RRR: 4.6; 95% CI 2.2–9.5; *p* < 0.001) and who were younger (RRR: 2.7; 95% CI 1.4–5.3; *p* = 0.003) were more likely to give birth at home with a SBA than at health facilities. Women whose child had a birth order of 1 (RRR: 0.22; 95% CI 0.96–0.52; *p* = 0.001) or 2 (RRR: 0.29; 95% CI 0.14–0.59; *p* = 0.001) were less likely to deliver at home with a SBA relative to having a facility delivery while women residing in rural areas (RRR: 2.1; 95% CI 1.2–3.8; *p* = 0.14) were more likely to have deliveries at home attended by a SBA than at a health facility.

**Table 2:** Factors associated with presence of a skilled birth attendant during delivery

Variables	Presence of skilled birth attendant at delivery					
	At home by unskilled birth attendant *N* = 58		At home by skilled birth attendant *N* = 111		At health facilities ^a^ *N* = 307	
	n (%)	RRR (95%CI)	n (%)	RRR (95%CI)	n (%)	RRR
Exposure to intervention						
No	35/266 (13%)	1.6 (0.84–2.9)	57/266 (21%)	1.2 (0.71–1.9)	174/266 (66%)	1.0
Yes ^a^	23/210 (11%)	1.0	54/210 (26%)	1.0	133/210 (63%)	1.0
Age						
< 25	32/277 (11%)	1.4 (0.64–3.0)	66/277 (24%)	2.7 (1.4–5.3) **	179/277 (65%)	1.0
25 or more ^a^	26/199 (13%)	1.0	45/199 (23%)	1.0	128/199 (64%)	1.0
Education						
No or primary education	35/186 (19%)	12.5 (3.5–45.0)* *	61/186 (33%)	4.6 (2.2–9.5)* *	90/186 (48%)	1.0
Junior secondary	20/152 (13%)	6.4 (1.8–22.9)* *	31/152 (20%)	1.9 (0.94–3.9)	101/152 (67%)	1.0
Secondary ^a^	3/138 (2%)	1.0	19/138 (14%)	1.0	116/138 (84%)	1.0
Income						
< 10,000BDT	40/223 (18%)	2.6 (1.3–5.1) *	61/223 (27%)	1.3 (0.77–2.2)	122/223 (55%)	1.0
10,000 BDT or above ^a^	18/253 (7%)	1.0	50/253 (20%)	1.0	185/253 (73%)	1.0
Birth order						
1	22/203 (11%)	0.71 (0.25–2.0)	41/203 (20%)	0.22 (0.96–0.52) *	140/203 (69%)	1.0
2	23/708 (13%)	0.81 (0.33–1.9)	36/178 (20%)	0.29 (0.14–0.59) *	119/178 (67%)	1.0
3 or more ^a^	13/95 (14%)	1.0	34/95 (36%)	1.0	48/95 (50%)	1.0
Residence						
Rural	25/215 (11%)	1.4 (0.72–2.9)	53/215 (25%)	2.1 (1.2–3.8) *	137/215 (64%)	1.0
Urban ^a^	33/261 (13%)	1.0	58/261 (22%)	1.0	170/261 (65%)	1.0
Districts						
District A	11/62 (18%)	12.1 (3.7–40.1) **	28/61 (46%)	11.3 (4.6–27.6) **	22/61 (36%)	1.0
District B	15/116 (13%)	2.2 (0.83–5.9)	11/116 (77%)	0.38 (0.16–0.91) *	90/116 (10%)	1.0
District C	17/115 (15%)	4.5 (1.6–12.3) **	32/115 (28%)	2.5 (1.2–5.2) *	66/115 (57%)	1.0
District D	7/62 (11%)	2.3 (0.65–8.2)	16/62 (26%)	2.2 (0.86–5.6)	39/62 (63%)	1.0
District E ^a^	8/122 (7%)	1.0	24/122 (20%)	1.0	90/122 (74%)	1.0

^a^delivery at health facilities, exposure to intervention, age 25 years or more, secondary education, income above 10,000 BDT, birth order 3 or more, urban residence and district E are reference category and RRR is expressed as 1.0. Significance is expressed as * when *p* < 0.05 and ** at *p* < 0.005

Women from district A were more likely to deliver at home with an unskilled birth attendant (RRR: 12.1; 95% CI 3.7–40.1; *p* < 0.001) or by a SBA (RRR: 11.3; 95% CI 4.6–27.6; *p* < 0.001) than at health facilities (Table 2). Similarly respondents from district C were more likely to have births at home with (RRR: 2.5; 5% CI 1.2–5.2; *p* = 0.014) or without a SBA (RRR: 4.5; 95% CI: 1.6–12.3; *p* = 0.003) while women from district B (RRR: 0.38; 95% CI 0.16–0.91; *p* = 0.03) were less likely to deliver at home with a SBA than at health facilities.

Most women (*n* = 443, 93%) fed their baby colostrum (Table 3) immediately after birth; the remaining 7% (*n* = 33) fed sweetened water, honey or mustard oil. Women’s exposure to mobile messages had no significant association with “breast feeding immediately post birth”. Respondents from districts A & D (OR: 9.6; 95% CI 2.4–37.6; *p* = 0.004), C (OR: 5.6; 95% CI 1.7–18.3; *p* = 0.001) and E (OR: 3.6; 95% CI 1.4–9.4; *p* = 0.007) had higher odds of initiating breastfeeding right after birth relative to district B. Women’s age, education, income, birth order, place of residence and facility based delivery were not significantly associated with colostrum feeding (Table 3).

**Table 3:** Breast milk feeding practice right after birth and associated factors

Variables	Fed breast milk after birth (*N* = 476)		
	Yes	No	OR (95% CI)
	n (%)	n (%)	
Exposure to intervention			
No ^a^	245 (92%)	21 (8%)	1.0
Yes	198 (94%)	12 (6%)	1.1 (0.51–2.5)
Age			
< 25 ^a^	253 (91%)	24 (9%)	1.0
25 or more	190 (96%)	9 (4%)	1.5 (0.54–3.6)
Education			
No or primary education	173 (93%)	13 (7%)	0.93 (0.32–2.6)
Junior secondary	140 (92%)	12 (8%)	0.76 (0.27–2.1)
Secondary ^a^	130 (94%)	8 (6%)	1.0
Income			
< 10,000BDT ^a^	204 (92%)	19 (8%)	1.0
10,000 BDT or above	239 (96%)	14 (5%)	1.9 (0.86–4.4)
Birth Order			
1	186/203 (92%)	17/203 (8%)	0.32 (0.07–1.4)
2	165/178 (93%)	13/178 (7%)	0.38 (0.09–1.5)
3 or more ^a^	92/95 (97%)	3/95 (3%)	1.0
Residence			
Rural ^a^	196/215 (91%)	19/215 (9%)	1.0
Urban	247/261 (95%)	14/261 (5%)	1.3 (0.56–2.9)
District			
District A + D	120/123 (98%)	3/123 (2%)	9.6 (2.4–37.6) **
District B ^a^	98/116 (85%)	18/116 (15%)	1.0
District C	111/115 (96%)	4/115 (4%)	5.6 (1.7–18.3) **
District E	114/122 (93%)	8/122 (7%)	3.6 (1.4–9.4) *
Facility based delivery			
No ^a^	157/169 (93%)	12/169 (7%)	1.0
Yes	286/307 (93%)	21/307 (7%)	1.4 (0.63–3.5)

^a^no-exposure to intervention, age 25 years or less, secondary education, income below 10,000 BDT, birth order 3 or more, rural residence and district B and delivery not at health facility are reference category and OR is expressed as 1.0. Significance is expressed as * when *p* < 0.05 and ** at *p* < 0.005. As all women in one district breastfed within 1 h of birth, this district had been combined with another for this analysis (A + D)

Overall, 62% (*n* = 294) of women bathed the baby 72 h or more after delivery, while 17% (*n* = 80) bathed babies in the first 48 h and 21% (*n* = 100) between 48 and 72 h. Women’s exposure to messages during pregnancy was not associated with bathing within the first 48 h, relative to delaying for at least 72 h, at the 5% level, but was significant at the 10% level (RRR 1.7; 95% CI 0.93–3.0; *p* = 0.083) in the adjusted regression model (Table 4). Younger (<25 years) women (RRR: 2.8; 95% CI 1.4–5.9; *p* = 0.005), were more likely to bathe their baby within the first 2 days relative to after 72 h. Women who did not deliver at a health facility were more likely to bathe their babies within 0–47 h (RRR: 4.8; 95% CI 2.6–9.1; *p* < 0.001) or within 48–72 h (RRR: 2.4; 95% CI 1.3–4.2; *p* = 0.003) than after 72 h. Women having their first (RRR: 0.3; 95% CI 0.1–0.8; *p* = 0.025) or second child (RRR: 0.3; 95% CI 0.1–0.8; *p* = 0.010) were less likely to bathe their babies within the first 2 days, relative to after 3 days, than women having at least their third child. Women from district B were more likely to bathe their babies within the first 48 h (RRR: 2.9; 95% CI 1.4–6.1; *p* = 0.004) or within 48–72 h (RRR: 3.4; 95% CI 1.6–7.1; *p* = 0.001), while those from district D were less likely to bathe their babies within the first 2 days (RRR: 0.20; 95% CI 0.05–0.74; *p* = 0.016) and those from district A were more likely to bathe within 48–72 h (RRR: 3.2; 95% CI 1.3–7.6; *p* = 0.008), relative to after 72 h, than women from district E.

**Table 4:** Factors associated with timing of first bath of new-born baby

Variables	0–47 h (*N* = 80)		48–72 h (*N* = 100)		72+ hours ^a^ (*N* = 294)	
	n (%)	RRR (95% CI)	n (%)	RRR (95% CI)	n (%)	RRR
Exposure to intervention						
No	53/265 (20%)	1.7 (0.93–3.0)	55/265 (21%)	1.2 (0.72–1.9)	156/265 (59%)	1.0
Yes ^a^	27/209 (13%)	1.0	45/209 (21%)	1.0	137/209 (63%)	1.0
Age						
< 25	52/277 (19%)	2.8 (1.4–5.9) *	63/277 (23%)	1.6 (0.8–3.0)	161/277 (58%)	1.0
25 or more ^a^	28/197 (14%)	1.0	37/197 (19%)	1.0	132/197 (67%)	1.0
Education						
No or primary education	38/185 (20%)	1.3 (0.59–3.1)	42/185 (23%)	1.1 (0.58–2.2)	105/185 (57%)	1.0
Junior secondary	29/152 (19%)	1.6 (0.75–3.6)	28/152 (18%)	0.93 (0.49–1.6)	95/152 (63%)	1.0
Secondary ^a^	13/137 (10%)	1.0	30/137 (22%)	1.0	93/137 (68%)	1.0
Income						
< 10,000BDT	43/223 (19%)	1.3 (0.70–2.3)	45/223 (20%)	0.93 (0.54–1.6)	135/223 (61%)	1.0
10,000 BDT or above ^a^	37/251 (15%)	1.0	55/251 (22%)	1.0	158/251 (63%)	1.0
Birth Order						
1	31/203 (15%)	0.34 (0.13–0.87) *	49/203 (24%)	0.83 (0.35–1.9)	123/203 (61%)	1.0
2	25/176 (14%)	0.34 (0.14–0.77) *	31/176 (18%)	0.63 (0.30–1.3)	120/176 (68%)	1.0
3 or more^a^	24/95 (25%)	1.0	20/95 (21%)	1.0	51/95 (54%)	1.0
Residence						
Rural	43/214 (20%)	0.80 (0.43–1.5)	41/214 (19%)	0.79 (0.46–1.4)	130/214 (61%)	1.0
Urban ^a^	37/260 (14%)	1.0	59/260 (23%)	1.0	164/260 (63%)	1.0
Districts						
District A	11/61 (18%)	0.75 (0.25–2.2)	28/61 (46%)	3.2 (1.3–7.6) *	22/61 (36%)	1.0
District B	15/116 (13%)	2.9 (1.4–6.1) *	11/116 (9%)	3.4 (1.6–7.1) **	90/116 (78%)	1.0
District C	17/115 (15%)	0.50 (0.21–1.2)	32/115 (28%)	1.0 (0.45–2.3)	66/115 (57%)	1.0
District D	7/62 (11%)	0.20 (0.05–0.74) *	16/62 (26%)	0.71 (0.26–1.9)	39/62 (63%)	1.0
District E ^a^	8/122 (6%)	1.0	24/122 (20%)	1.0	90/122 (74%)	1.0
Facility based delivery						
No	46/168 (27%)	4.8 (2.6–9.1) **	45/168 (27%)	2.4 (1.3–4.2) **	77/168 (46%)	1.0
Yes ^a^	34/306 (11%)	1.0	55/306 (18%)	1.0	217/306 (71%)	1.0

^a^bathing 72+ hours, exposure to intervention, age 25 years or more, secondary education, income above 10,000 BDT, birth order 3 or more, urban residence and district E are reference category and RRR is expressed as 1.0. Significance is expressed as * at *p* < 0.05 and ** at *p* < 0.005.

More than half the women (*n* = 273, 57%) did not have postnatal care (PNC) visits within 42 days after childbirth. The rest reported having 1 (*n* = 79, 17%), 2 (*n* = 54, 11%), 3 (*n* = 34, 7%) or 4 visits (*n* = 36, 8%). Negative binomial analysis showed that women’s exposure to mobiIe phone messages during pregnancy had no association with the number of PNC visits at the 5% significance level (IRR: 1.2; 95%CI 0.94–1.6; *p* = 0.117), as shown in Table 5. Women who had a facility based delivery had a 52% higher rate of PNCs than women who birthed at home (IRR: 1.52; 95% CI: 1.1–2.1; *p* < 0.001). Urban women had 63% higher rate of PNC visits than rural women (IRR: 1.63; 95% CI 1.2–2.2; *p* = 0.002). Districts B (RRR: 0.57; 95% CI 0.36–0.91; *p* = 0.018), C (RRR: 0.41; 95% CI 0.26–0.64; *p* < 0.001), D (RRR: 0.12; 95%CI 0.06–0.23; *p* < 0.001) and E (RRR: 0.30; 95% CI 0.18–0.50; *p* < 0.001) had lower rates of PNC visits than District A.

**Table 5:** Factors associated with frequency of postnatal care visits

Variables	PNC in 42 days					
	No visits (*n* = 273)	1 visit (*n* = 74)	2 visits (*n* = 54)	3 visits (*n* = 34)	4 visits (*n* = 36)	IRR (95%CI)
Exposure to intervention						
No ^a^	158/266 (60%)	48/266 (18%)	32/266 (12%)	11/266 (4%)	17/266 (6%)	1.0
Yes	115/210 (55%)	31/210 (15%)	22/210 (10%)	23/210 (11%)	19/210 (9%)	1.2 (0.94–1.6)
Age						
< 25 ^a^	163/277 (59%)	42/277 (15%)	30/277 (11%)	20/277 (7%)	22/277 (8%)	1.0
25 or more	110/199 (55%)	37/199 (19%)	24/199 (12%)	14/199 (7%)	14/199 (7%)	0.82 (0.57–1.7)
Education						
No or primary education ^a^	121/186 (65%)	26/186 (14%)	21/186 (11%)	11/186 (6%)	7/186 (4%)	1.0
Junior secondary	81/152 (53%)	32/152 (21%)	18/152 (12%)	11/152 (7%)	10/152 (7%)	1.3 (0.90–1.8)
Secondary	71/138 (51%)	21/138 (15%)	15/138 (11%)	12/138 (9%)	19/138 (14%)	1.4 (0.96–2.1)
Income						
< 10,000BDT ^a^	141/223 (63%)	35/223 (16%)	24/223 (11%)	9/223 (4%)	14/223 (6%)	1.0
10,000 BDT or above	132/253 (52%)	44/253 (17%)	30/253 (12%)	25/253 (10%)	22/253 (9%)	1.2 (0.87–1.8)
Birth Order						
1 ^a^	117/203 (58%)	33/203 (16%)	23/203 (11%)	12/203 (6%)	18/203 (9%)	1.0
2	106/178 (60%)	25/178 (14%)	19/178 (11%)	13/178 (7%)	15/178 (8%)	1.1 (0.9–1.8)
3 or more	50/95 (53%)	21/95 (22%)	12/95 (13%)	9/95 (9%)	3/95 (3%)	1.4 (0.9–2.4)
Residence						
Rural^a^	140/215 (65%)	37/215 (17%)	16/215 (7%)	12/215 (6%)	10/215 (5%)	1.0
Urban	133/261 (51%)	42/261 (16%)	38/261 (15%)	22/261 (8%)	26/261 (10%)	1.63 (1.2–2.2) **
District						
District A ^a^	22/61 (36%)	3/61 (5%)	13/61 (21%)	8/61 (13%)	15/61 (25%)	1.0
District B	46/116 (40%)	29/116 (25%)	21/116 (18%)	11/116 (9%)	9/116 (8%)	0.57 (0.36–0.91) *
District C	70/115 (61%)	17/115 (15%)	11/115 (10%)	9/115 (7%)	8/115 (7%)	0.41 (0.26–0.64) **
District D	48/62 (77%)	11/62 (18%)	2/62 (3%)	1/62 (2%)	0/62 (0%)	0.12 (0.06–0.23) **
District E	87/122 (71%)	19/122 (16%)	7/122 (6%)	5/122 (4%)	4/122 (3%)	0.30 (0.18–0.50) **
Facility based delivery						
No ^a^	113/169 (67%)	20/169 (12%)	13/169 (8%)	11/169 (6%)	12/169 (7%)	1.0
Yes	160/307 (52%)	59/307 (19%)	41/307 (13%)	23/307 (8%)	24/307 (8%)	1.52 (1.2–2.1) *

^a^no-exposure to intervention, age 25 years or less, no or primary education, income below 10,000 BDT, birth order 1, rural residence and district A are reference category and IRR is expressed as 1.0. Significance is expressed as * at *p* < 0.05 and ** at *p* < 0.005

## Discussion

We have analysed the impact of mobile phone messages (voice or text) among women who were recruited in the Aponjon service during their pregnancy or the postnatal period. Our study has two strengths: one, it identifies the relationship between timing of receipt of mobile phone messages and several outcomes and two, we consider the impact of other covariates on health behaviour of women, an important consideration when designing a mobile phone based health information service. While in the model adjusted for all covariates (age, education, income, place of residence, birth order, facility delivery) and district, the timing of receipt of mobile phone messages was not associated with timing of first bath at the 5% level, a *p* value of 0.083 indicates that there may be some evidence of an association for bathing within 48 h versus after 3 days, particularly given that the study was only powered to detect moderate to large associations. Delaying the “timing of first bath” is an important contribution to neonatal care as early bathing is a longstanding cultural practice often found in ultra-poor households in which relatives bathe the baby within a few hours after birth even on a cold night to respond to perceptions of birth-related pollution [24–26]. This practice contributes to neonatal mortality by leading to a drop in the baby’s body temperature [24, 27, 28]. The “warm chain” guideline suggests that birthing should occur in a warm delivery room, that the baby is immediately dried and wrapped, has skin-to-skin contact with mother, is breastfed immediately after birth and that bathing is delayed until the second or third day when the baby is healthy and has a normal temperature [5]. In our study, bathing 72 h or more after birth was most common among older women (age > 25 years), women with one or two children and women who had a facility based delivery. Timing of the first bath varied substantially among the study districts, with two districts in particular more likely to bathe their babies within 2 days, a finding consistent with national survey data [1]. This could be due to cultural belief and practice in that region which is beyond the scope of this analysis. This variation is worth exploring in further studies, as it may provide useful information on factors that influence the effectiveness of such messages.

The timing of exposure to mobile phone messages had no significant association with the rest of the outcome variables. Women from a privileged background (higher education and higher income), those who lived in urban areas and those who had their first or second child were more likely to deliver at a health facility; a finding consistent with previous studies [1, 29–32]. It is likely that poorer women and their families choose untrained traditional birth attendants for deliveries to minimise expenses [32–34]. Previous studies have shown that the older a woman is at marriage the more likely she is to use a facility for delivery which correlates with our finding that younger mothers are more likely to give birth at home with a SBA than at a health facility [35].

It was common among the study respondents to initiate breastfeeding in the first hour after delivery irrespective of their socio-economic status. However, there was some variation across districts, possibly due to differences in cultural practices and beliefs.

Though number of PNC visits is gradually increasing in Bangladesh, more than 60% of mothers and new born babies do not get PNC within the first 2 days of birth by a medically trained provider [1]. In some cultural contexts women are secluded after childbirth and prohibited from seeing anyone outside family members except when very ill, when a provider is summoned to the home [36, 37]. Previous research suggests that the family’s lack of knowledge about symptoms of illness, concern over medical costs, social disparity and reliance on non-medical healers has delayed seeking proper healthcare and contributes to maternal and child mortality [31, 38–41]. Our findings confirm existing knowledge that women’s higher education and urban residence have a pertinent role in PNC visit frequencies [42]. Facility based delivery was an independent factor for the number of PNC visits in our study. It is evident from other studies that facility based delivery has a significant role in initiating the recommended first PNC visit on the same or next day, which may be reinforced by hospital staff after delivery [1, 4, 43, 44].

As facility delivery is an independent factor for the outcomes delayed bathing and PNC visits, we raise the question whether the messages that refer to practices that are already widely adopted should be de-emphasised and the service should concentrate on messages during pregnancy such as “planning facility based delivery over home delivery” which would contribute to other best practices. It is known from other research that women’s higher education and wealth are pivotal in delaying marriage and conception [45]. Since educated women, older mothers and women with one or two children reinforced good practices regarding delivery place, delaying bathing and the number of PNC visits, we anticipate national efforts aimed at improving indicators of women’s autonomy (e.g. women’s education and income) will lead to maternal and child health improvements over time. Mobile messaging, especially voice messaging which does not require participants to be literate, may be one route to address these disparities.

### Limitations

As this was an observational rather than a randomized study, the study did not have a formal control group and instead we categorised users based on their inclusion in the service at different time points during the pregnancy or the post-natal period, which may limit generalizability of the analysis and causation cannot be ascertained. Due to the sample size our ability to observe an association between early messages and outcomes was limited. The power of our study was based on a moderate to large association, hence small differences in the outcomes associated with the timing of receipt of the Aponjon messages could not be captured. In addition we cannot exclude the possibility of study participants being exposed to health related information from the health workers during their routine antenatal care visits at home. We do not have information on the partner organizations’ health promotion activities in their catchment area or the time of inclusion of the districts in the service which could help us to understand the difference in the outcomes. The study relies on self-report of practices which can introduce bias and problems of recall and may lead to overestimation of performance [46]. District is a potential confounder for the study due to the recruitment strategy of the service where health workers from different partner organizations recruited subscribers in different districts at different point of service year. The association between district and some of the outcomes may reflect cultural practices and other factors not captured in our study. There are many contextual factors which contribute to delivery practices which the survey did not attempt to capture, such as information on the experiences of families regarding their access to local health facilities, availability of qualified staff and equipment at local facilities, and current and previous pregnancy complications [29, 35, 47–49]. Nevertheless, this study contributes to our understanding of what can be achieved by a service such as Aponjon which is one of the first to scale up in a resource-limited country. Our study points to the need for larger studies and randomised designs to overcome the limitations encountered in this study.

## Conclusion

Our results indicate that mobile phone text or voice messages may delay timing of first bath within the first 48 h after birth. Although a formal control group and larger sample size would produce more generalizable results and potentially allow detection of small to moderate associations, our study suggests that low-cost mobile phone educational service may work as catalysts in improving maternal and child health behaviour in resource-limited settings. Our study demonstrates yet again that addressing maternal and child health disparities requires investment in what we already know works, such as training health staff, building health facilities and educating women. Mobile phone messages may be a promising addition to ensure that such interventions achieve maximum impact. We plan to undertake additional research in the Aponjon sample by exploring participants’ perceptions and experiences of the Aponjon healthcare service with the aim of providing information to help develop a national framework for an integrated health system.

## Additional file

Additional file 1: Questionnaire used during the survey of Dnet to interview Aponjon subscribers. (PDF 939 kb)

## Abbreviations

ANU: The Australian National University
BDT: Bangladeshi Taka (currency)
DERC: Delegated Ethics Review Committee
Dnet: Implementing agency of Aponjon service in Bangladesh
IRB: Institutional Review Board
mHealth: Mobile Health as in health practice supported by mobile phones
MOHFW: Ministry of Health and Family Welfare
NGO: Non-Government Organizations
PNC: Postnatal Care
SBA: Skilled Birth Attendant
USAID: The United States Agency for International Development
USD: The United States Dollar (currency)

## References

[1] Bangladesh Demographic and Health Survey 2014. Dhaka, Bangladesh, and Rockville, Maryland, USA: NIPORT, Mitra and Associates, and ICF International. 2016. https://dhsprogram.com/pubs/pdf/FR311/FR311.pdf. Accessed 15 Nov 2016.

[2] Health, population and nutrition sector development programme (HPNSDP) July 2011–June 2016. Dhaka: Ministry of Health and Family Welfare Government of the People’s Republic of Bangladesh. 2012. http://www.nationalplanningcycles.org/sites/default/files/country_docs/Bangladesh/bangladesh_hpnsdp_2011-2016.pdf. Accessed 15 Nov 2016.

[3] Care in normal birth: a practical guide. Geneva: World Health Organization. 1996. http://apps.who.int/iris/bitstream/10665/63167/1/WHO_FRH_MSM_96.24.pdf. Accessed 15 Nov 2016.

[4] Postnatal care of mothers and new-borns: highlights from the World Health Organizations 2013 guidelines. World Health Organization and Jhpiego. 2015. http://www.who.int/maternal_child_adolescent/publications/WHO-MCA-PNC-2014-Briefer_A4.pdf. Accessed 15 Nov 2016.

[5] Thermal protection of the new-born: a practical guide. Geneva: World Health Organization. 1997. http://apps.who.int/iris/bitstream/10665/63986/1/WHO_RHT_MSM_97.2.pdf. Accessed 15 Nov 2016.

[6] Mullany LC. Neonatal hypothermia in low-resource settings. Semin Perinatol. 2010; doi:10.1053/j.semperi.2010.09.007. 10.1053/j.semperi.2010.09.007 PMC3001630 21094417

[7] Garofalo R. Cytokines in human milk. J Pediatr.2010; doi:10.1016/j.jpeds.2009.11.019. 10.1016/j.jpeds.2009.11.019 20105664

[8] Walker WA, Iyengar RS. Breast milk, microbiota, and intestinal immune homeostasis. Pediatr Res. 2015; doi:10.1038/pr.2014.160. 10.1038/pr.2014.160 25310762

[9] WHO global observatory for eHealth. mHealth New horizons for health through mobile technologies. Switzerland: World Health Organization. 2011. http://www.who.int/goe/publications/goe_mhealth_web.pdf. Accessed 15 Nov 2016.

[10] Bangladesh telecommunication regulatory commission. Joint ITU-WHO meeting on “National eHealth strategy development: Bangladesh perspective. Geneva: World Health Organization. 2012. https://www.itu.int/ITU-D/cyb/events/2012/e-health/Nat_eH_Dev/Session%203/Bangladesh/WHO-ITU%20Presentation-Rev%202.0%20(1).pdf. Accessed 15 Nov 2016.

[11] Health information system & eHealth. Health, population and nutrition sector development program 2011–16.Dhaka: Directorate General of Health services, Ministry of Health and family welfare. 2011. http://www.mohfw.gov.bd/index.php?option=com_content&view=article&id=166&Itemid=150&lang=en. Accessed 15 Nov 2016.

[12] Rajan R, Raihan A, Alam M, Agarwal S, Ahsan A, Bashir R (2013). MAMA ‘Aponjon’ formative research report.

[13] Aponjon News Issue 1 October–December 2012. http://www.aponjon.com.bd/pdf_view/aponjon_newsletter_55ed2cfa132d5.pdf. Accessed 15 Nov 2016.

[14] Hall CS, Fottrell E, Wilkinson S, Byass P. Assessing the impact of mHealth interventions in low and middle income countries what has been shown to work? Glob Health Action. 2014; doi:10.3402/gha.v7.25606. 10.3402/gha.v7.25606 PMC4216389 25361730

[15] Watterson JL, Walsh J, Madeka I. Using mHealth to Improve Usage of Antenatal Care, Postnatal Care, and Immunization: A Systematic Review of the Literature. Biomed Res Int. 2015; doi:10.1155/2015/153402. 10.1155/2015/153402 PMC4561933 26380263

[16] Lund S, Nielsen BB, Hemed M, Boas IM, Said A, Said K, et al. Mobile phones improve antenatal care attendance in Zanzibar: a cluster randomized controlled trial. BMC Preg Childbirth. 2014; doi:10.1186/1471-2393-14-29. 10.1186/1471-2393-14-29 PMC3898378 24438517

[17] Gallegos D, Russell-Bennett R, Previte J, Parkinson J. Can a text message a week improve breastfeeding? BMC Pregnancy Childbirth. 2014; doi:10.1186/s12884-014-0374-2. 10.1186/s12884-014-0374-2 PMC4237760 25369808

[18] Smith C, Ngo TD, Gold J, Edwards P, Vannak U, Sokhey L. Effect of a mobile phone-based intervention on post-abortion contraception: a randomized controlled trial in Cambodia. Bull World Health Organ. 2015; doi:10.2471/BLT.15.160267. 10.2471/BLT.15.160267 PMC4669734 26668436

[19] Evans WD, Wallace JL, Snider J.Pilot evaluation of the text4baby mobile health program. BMC Pub Health. 2012; doi:10.1186/1471-2458-12-1031. 10.1186/1471-2458-12-1031 PMC3570294 23181985

[20] Tamrat T, Kachnowski S. Special delivery: an analysis of mHealth in maternal and new-born programs and their outcomes around the world. Matern Child Health J. 2012; doi:10.1007/s10995-011-0836-3. 10.1007/s10995-011-0836-3 21688111

[21] Lee HS, Nurmatov UB, Nwaru BI, Mukherjee M, Grant L, Pagliari C. Effectiveness of mHealth interventions for maternal, new-born and child health in low– and middle–income countries: Systematic review and meta–analysis. J Glob Health. 2016; doi:10.7189/jogh.06.010401. 10.7189/jogh.06.010401 PMC4643860 26649177

[22] Ahmed T, Lucas H, Khan AS, Islam R, Bhuiya A, Iqbal M. eHealth and mHealth initiatives in Bangladesh: a scoping study. BMC Health Serv Res. 2014; doi:10.1186/1472-6963-14-260. 10.1186/1472-6963-14-260 PMC4072608 24934164

[23] Tswae M, Moto A, Netshivhera T, Ralesego L, Nyathi C, Susuman AS. Factors influencing the use of maternal healthcare services and childhood immunization in Swaziland. Int J Equity Health. 2015; doi:10.1186/s12939-015-0162-2. 10.1186/s12939-015-0162-2 PMC4391603 25889973

[24] Winch PJ, Alam MA, Akther A, Afroz D, Ali NA, Ellis AA, et al. Local understandings of vulnerability and protection during the neonatal period in Sylhet district, Bangladesh: a qualitative study**.** Lancet. 2005; doi:10.1016/S0140-6736(05)66836-5. 10.1016/S0140-6736(05)66836-5 16084256

[25] Darmstadt GL, Syed U, Patel Z, Kabir N (2006). Review of domiciliary new-born-care practices in Bangladesh. J Health Popul Nutr.

[26] Blanchet T (1984). Meanings and rituals of birth in rural Bangladesh: women, pollution and marginality.

[27] Lunze K, Bloom DE, Jamison DT, Hamer DH. The global burden of neonatal hypothermia: systematic review of a major challenge for new-born survival. BMC Med. 2013; doi:10.1186/1741-7015-11-24. 10.1186/1741-7015-11-24 PMC3606398 23369256

[28] Kumar V, Shearer JC, Kumar A, Darmstadt GL. Neonatal hypothermia in low resource settings: a review. J Perinatalogy. 2009; doi:10.1038/jp.2008.233. 10.1038/jp.2008.233 19158799

[29] Bangladesh Maternal Mortality and Healthcare Survey 2010. Dhaka, Bangladesh: NIPORT, MEASURE Evaluation, and ICDDR,B; 2012. https://www.measureevaluation.org/resources/publications/tr-12-87.Accessed 15 Nov 2016.

[30] Kamal SM. Safe motherhood practices among women in Bangladesh. Healthcare Women Int.2012; doi:10.1080/07399332.2012.655387. 10.1080/07399332.2012.655387 22827729

[31] Chowdhury RI, Islam MA, Gulshan J, Chakraborty N. Delivery complications and healthcare-seeking behavior: the Bangladesh demographic health survey, 1999-2000. Health Soc Care Community. 2007; doi:10.1111/j.1365-2524.2006.00681.x. 10.1111/j.1365-2524.2006.00681.x 17444989

[32] Dalal K, Shabnam J, Andrews-Chavez J, Mårtensson LB, Timpka T (2012). Economic empowerment of women and utilization of maternal delivery care in Bangladesh. Int J Prev Med.

[33] Choudhury N, Ahmed SM. Maternal care practices among the ultra poor households in rural Bangladesh: a qualitative exploratory study. BMC Preg Childbirth.2011; doi:10.1186/1471-2393-11-15. 10.1186/1471-2393-11-15 PMC3056829 21362164

[34] Choudhury N, Moran AC, Alam MA, Ahsan KZ, Rashid SF, Streatfield PK. Beliefs and practices during pregnancy and childbirth in urban slums of Dhaka, Bangladesh. BMC Pub Health. 2012; doi:10.1186/1471-2458-12-791. 10.1186/1471-2458-12-791 PMC3532223 22978705

[35] Edmonds JK, Paul M, Sibbley L. Determinants of place of birth decisions in uncomplicated childbirth in Bangladesh: An empirical study. Midwifery. 2012; doi:10.1016/j.midw.2011.12.004. 10.1016/j.midw.2011.12.004 PMC3472154 22884893

[36] Goodburn EA, Gazi R, Chowdhury M (1995). Beliefs and practices regarding delivery and postpartum maternal morbidity in rural Bangladesh. Stud Fam Plan.

[37] Moran AC, Winch PJ, Sultana N, Kalim N, Afzal KM, Koblinsky M, et al. Patters of maternal care seeking behaviours in rural Bangladesh. Trop Med Int Health. 2007; doi:10.1111/j.1365-3156.2007.01852.x. 10.1111/j.1365-3156.2007.01852.x 17596248

[38] Killewo J, Anwar I, Bashir I, Yunus M, Chakraborty J (2006). Perceived delay in healthcare-seeking for episodes of serious illness and its implications for safe motherhood interventions in rural Bangladesh. J Health Popul Nutr.

[39] Langlois EV, Miszurka M, Zunzunegui MV, Ghaffr A, Ziegler D, Karp I. Inequities in postnatal care in low-and middle-income countries: a systematic review and meta-analysis. Bull World Health Organ.2015; doi:10.2471/BLT.14.140996. 10.2471/BLT.14.140996 PMC4431556 26229190

[40] Koenig MA, Jamil K, Streatfield PK, Saha T, Al-Sabir A, Arifeen SE, et al. Maternal health and care-seeking behaviour in Bangladesh: Findings from a national survey. Int Fam Plan Perspect.2007; doi:10.1363/ifpp.33.075.07. 10.1363/3307507 17588851

[41] Mercer A, Haseen F, Huq NL, Uddin N, Khan MH, Larson CP. Risk factors for neonatal mortality in rural areas of Bangladesh served by a large NGO programme. Health Policy Plan.2006; doi:10.1093/heapol/czl024. 10.1093/heapol/czl024 16943220

[42] Shahabuddin AS, Delvaux T, Abouchadi S, Sarker M, De Brouwere V. Utilization of maternal health services among adolescent women in Bangladesh: A scoping review of the literature. Trop Med Int Health. 2015; doi:10.1111/tmi.12503. 10.1111/tmi.12503 25757880

[43] Baqui AH, Ahmed S, Arifeen SE, Darmstadt GL, Rosecrans AM, Mannan I, et al. Effect of timing of first postnatal care home visit on neonatal mortality in Bangladesh: a observational cohort study. BMJ. 2009; doi:10.1136/bmj.b2826. 10.1136/bmj.b2826 PMC2727579 19684100

[44] Persson EK, Fridlund B, Kvist LJ, Dykes A. Mothers’ sense of security in the first postnatal week: interview study. J Adv Nurs. 2011; doi:10.1111/j.1365-2648.2010.05485.x. 10.1111/j.1365-2648.2010.05485.x 20969617

[45] Nasrin SO, Rahman KMM. Factors affecting early marriage and early conception of women: A case of slum areas in Rajshahi City, Bangladesh. Int J Soc Anth. 2012; doi:10.5897/IJSA11.145.

[46] Tarrant RC, Younger KM, Sheridan-Pereira M, Kearney JM. Maternal health behaviours during pregnancy in an Irish obstetric population and their associations with socio-demographic and infant characteristics. Eur J Clin Nut. 2011; doi:10.1038/ejcn.2011.16. 10.1038/ejcn.2011.16 21364609

[47] Thaddeus S, Maine D (1994). Too far to walk: maternal mortality in context. Soc Sci Med.

[48] Sarker BK, Rahman M, Rahman T, Hossain J, Reichenbach L, Mitra DK. Reasons for preference of home delivery with traditional birth attendants (TBAs) in rural Bangladesh: a qualitative exploration. PLoS One. 2016; doi:10.1371/journal.pone.0146161. 10.1371/journal.pone.0146161 PMC4701391 26731276

[49] Shanmugavelan M, Alam M, Raihan A, Shoemacker E (2011). Mobile phones for millennium development goal: qualitative need analysis to overcome communication barriers to improve maternal health.

