# Supplementary material for: The impact of mobile phone based messages on maternal and child healthcare behaviour: a retrospective cross-sectional survey in Bangladesh
Source: BMC Health Serv Res. 2017 Jun 24;17:434. doi: 10.1186/s12913-017-2361-6 (PMC5482970; doi:10.1186/s12913-017-2361-6)
Supplement: Additional file 1: — Questionnaire used during the survey of Dnet to interview Aponjon subscribers. (PDF 939 kb) [file 12913_2017_2361_MOESM1_ESM.pdf]

Sample survey questionnaire

2014

New mother

Interview ID: NM___________ (3 digits)

Location __________________

Written consent for interview, photograph and audio recording:

Hi, my name is ___________ and I am involved in a mobile phone based service research study called Aponjon (a brand service under the initiative of Mobile Alliance for Maternal Action). This research is initiated by USAID and implemented by Dnet with support of outreach partners BRAC, Save the Children USA, NHSDP, Infolady, Ministry of Health and Family welfare (Government of Bangladesh) and PM's Access to Information services programme.

You are selected as a participant of this study as you are either pregnant or mother of an infant and had been registered for mobile based service for the last couple of months. I will ask you to participate in a survey that would include questions about how you feel about the service, what changes the service made/may make in your life, feedbacks regarding the service and compliance with the payment process of the service. The length of the survey will be about one hour.

There should be little or no risk involved in participating in this research. If any of the questions make you feel uncomfortable, you do not have to answer them. Your participation is voluntary, and you can leave the survey or withdraw from the study at any time without any consequences to you. We will audio record the survey so that we can have a good record of what everyone says. We will transcribe (write down) the information from the recording and erase the recording after writing the final draft.

We may also ask to take photos of you. The pictures may be published in reports and journal articles but would never include your name or name of this venue. Photos may be posted online in reports or the project website. After we take the photographs, you may view them and request that they not be used.

The information from this study may be used in the future for other studies on similar topics. You will not benefit directly from participating in this research. If you wish to participate in this research we have some gift items from this service as a token of your valuable time to the survey.

Any information that is obtained in connection with this study and that can be identified with you will remain confidential and will be disclosed only with your permission. The information will be kept in a secure location where only the researchers in this study will have access. We will replace your name with a code. Data from this study will be kept indefinitely.

Here is my card with my name, contact information, name of the research organization responsible for the research. Please feel free to contact me in future if you have any question related to the survey. You might also check out your right to participate in the survey, in that case I can provide you with the contact details to USAID.

Do you have any questions about this research? Yes/No (Tick as appropriate)

Are you willing to participate in the survey? Yes/No (Tick as appropriate)

Do you give us permission to take photographs of you? Yes/No (Tick as appropriate)

Thank you So much for your cooperation

____

Signature & Name of interviewee

____

Signature & Name of interviewer

Date:

Time of interview: Start End:

1. Interviewer's ID:

Background information

	Question	Answer	Instructions for field researcher
2.	Respondent's Name:<br>(Masked in the database)	Write here	
3.	Respondent's Registration ID<br><br>(Masked in the database)	Write here	
4.	Type of respondent	o New mother (2)	
5.	Cell number of respondent (Masked in the database)	Write here	
6.	Type of Aponjon messages received	o Text messages (1)<br>o Recorded voice messages (2)	
7.	If receives recorded messages, then when is the time they receive messages?	o Between 8 am -12 pm<br>o Between 12-4 pm<br>o Between 4-8 pm<br>o Between 8-11 pm	
8.	What is the payment method for the service?	o The service is free of charge<br>o Pays the regular service price	
9.	New mother's last menstruations' first date or date of birth of baby	Write here	
10.	Address of respondent	Division<br>District:<br>Sub-district/Upazilla:<br>Union:<br>Village:	
11.	Education level of new mother	o Never attended school (1)<br>o Primary education incomplete (2)<br>o Primary education complete (3)<br>o Junior secondary /grade 8 complete (4)<br>o Secondary education complete (5)<br>o Higher Secondary incomplete/grade 11 (6)<br>o Higher education complete	

		(7)<br>o Higher than HSC/More than 12 years of education (8)<br>o Others ________(66)	
12.	Education level of new mother's husband	o Never attended school (1)<br>o Primary education incomplete (2)<br>o Primary education complete (3)<br>o Junior secondary (level 8) complete (4)<br>o Secondary education complete (5)<br>o Higher Secondary incomplete/grade 11 (6)<br>o Higher education complete (7)<br>o Higher than HSC/More than 12 years of education (8)<br>o Others ________(66)	
13.	Occupation of new mother	o Housewife (1)<br>o student (2)<br>o Day laborer (3)<br>o Farm (4)<br>o Fisher woman (5)<br>o Driver –car/heavy vehicle (6)<br>o Small businessman (owner of local shop etc.) (7)<br>o Business man (8)<br>o Govt. emplyees (9)<br>o Private service holder(10)<br>o NGO worker (11)<br>o Garments worker (12)<br>o Others_______________(66)	
14.	Occupation of new mother's husband	o Unemployed (1)<br>o Student (2)<br>o Day laborer (3)<br>o Farmer (4)<br>o Fisherman(5)<br>o Rickshaw puller (6)<br>o Driver- CNG (7)<br>o Driver –Car/ Heavy vehicle (8)<br>o Small business man (9)<br>o Businessman (10)<br>o Govt. employee (11)<br>o Private service holder (12)<br>o NGO worker (13)	

		o Overseas worker (14)<br>o Others _____________(66)	
15.	Monthly family income (BDT)	o Below 5000 taka (1)<br>o 5001-10000 (2)<br>o 10001-20000 (3)<br>o 20001-50000 (4)<br>o 50001-100000 (5)<br>o 100000 or above (6)	
16.	Sanitary latrine present at residence?	o Yes (1)<br>o No (2)	
17.	Age of baby	o Below 28 at days (1)<br>o Below 3 months (2)<br>o Below 6 months (3)<br>o Below 9 months (4)<br>o Below 12 months (5)<br>o Above 12 months (6)	
18.	Age of new mother	o Below 15 years (1)<br>o 15-19 years (2)<br>o 20-24 years (3)<br>o 25-29 years (4)<br>o 30-34 years (5)<br>o 35 and above (6)	
19.	How many children she have?	o 1 (1)<br>o 2 (2)<br>o 3 (3)<br>o 4 (4)<br>o More than 4 (5)	
20.	What is the age difference between the last issue and this one?	Write here:	
20.	Aponjon service enjoyed by	o New mother alone (1)<br>o New mother and husband (2)<br>o New mother and others (3)<br>o Only husband (4)<br>o Only guardian (other than husband) (5)<br>o Others__________ (66)	
21.	She receives Aponon's	o Urban version(1)<br>o Rural version (2)	
22.	Aponjon service is received by	o Mother Alone (1)<br>o New mother and husband (2)<br>o New mother and guardian (other than husband) (3)<br>o Only husband (4)<br>o Only guardian (other than husband) (5)<br>o Others ___(66)	

23.	Reason for interviewing this guardian	o They received messages during pregnancy and now receiving for new mother and baby (1)<br>o They are receiving messages for new mother (2)<br>o They used to receive messages for new mother, but as the baby crossed first birthday, not receiving messages anymore (3)<br>o Others _____(66)	

Aponjon service related

	Questions	Answer	Instructions for field researcher
24.	Who enrolled you in this service?	o Health worker (1)<br>o From hospital (2)<br>o Brand promoter (3)<br>o New mother (4)<br>o Respondent (guardian) called or texted to 16227 (5)<br>o Others_______ (66)	
25.	Before enrollment, how did you find out this service?	o From health worker(1)<br>o From an Aponjon subscriber(2)<br>o From hospital(3)<br>o From Blue star clinics(4)<br>o From a brand promoter(5)<br>o Husband (6)<br>o From TV (7)<br>o From billboard(8)<br>o From newspaper advertisements(9)<br>o From SMS in my phone (10)<br>o I received calls about this service(11)<br>o From leaflet (12)<br>o I don't know (98)<br>o Others________ (66)	
26.	Do you currently receive Aponjon messages?	o Yes (1)<br>o No (2)<br>o Received it previously, but not anymore (3)<br>o Others________(66)	
27.	Which messages did you receive? Was these messages intended for you?	o The respondent received messages customized for guardians(1)<br>o The respondent received new mother's messages as his/her cell number was	

		subscriberd as primary recipient (2)<br>o For someone else in the house, as the cell phone is shared among family members(3)<br>o Neighbor's - on request phone(4)<br>o Others_____ (66)	
28.	How many messages you receive weekly now?	o 0 (1)<br>o 1 (2)<br>o 2 (3)<br>o 3 (4)<br>o More than 3 (5)<br>o Never received any messages (6)<br>o I received messages but did not notice when it came as I was not aware I was enrolled in the service (7)<br>o I don't receive the messages as it drops phone balance(8)<br>o Others __(66)	
29.	Who receives messages?	o New mother herself (1)<br>o Some one else other than new mother (who owns the phone) (2)<br>o New mother or some one from the house (shared family phone) (3)	
30.	Do you encounter any problem to receive the messages?	o Yes (1)<br>o No (2)<br>o Don't know (98)<br>o Others _____(66)	If yes, go to next question no. 31
31.	If yes, what kind of problem did you encounter?	o The service is pricy (1)<br>o I can't listen to the messages myself as the call comes when the cell phone is not with me (2)<br>o Don't receive messages anymore due to loss pf phoneset/ sim card (3)<br>o I can't read the transliterated text messages (4)<br>o In the recorded voice messages, the messages are said with hurry , difficult to get the information (5)<br>o The baby did not survice, but still receiving messages which is annoying (6)<br>o There is nothing new in this information (7)<br>o The weekly messages are not	

		according to new mother's current gestational state (8)<br>o I can't hear anything after receiving the phone call (problem due to zero balance in phone) (9)<br>o I can't hear after receiving the phone call (mobile network is weak in that area)<br>o Others___________ (66)	
32.	Can you remember the short code which provides you the messages?	o Could tell the full shortcode (1)<br>o Could tell the shortcode partially(2)<br>o Could not tell the shorcode(3)	

Knowledge and practices on delivery and new born care

	Questions	Answer	Instructions for field researchers
33.	Where was the baby born?	○ Hospital/clinic/ health facilities (1)<br>○ Home (2)	If born at hospital/clinic/health facilities than skip to Q-37
34.	(If yes) Who assisted the delivery?	○ Trained birth attendant<br>○ Untrained birth attendant (Mother, mother-in-law, neighbor etc. who do not have a training)<br>○ Others_______(66)	For deliveries at home
35.	What equipment did the birth attendant use during delivery?	○ Safe delivery kit (1)<br>○ Clean blade, rubber, scissors (2)<br>○ Anything that was available during that time (3)<br>○ Others___ (66)	For deliveries at home
36.	How did you know about using safe delivery kit	o Only Aponjon (1)<br>o Aponjon and other sources (2)<br>o Other sources (3)<br>o Don't know (98)<br>o Others___(66)	For deliveries at home
37.	After delivery, did new mother go for physical	o Yes (1)<br>o No (2)	If answer is "No" skip to Q-45

	checkup?		
38.	Reason for the physical check up	o For mother's health (1)<br>o For baby's health (2)<br>o Others_____ (66)	
39.	Who advised you to go for physical visits?	o I knew about this (1)<br>o Husband (2)<br>o Elderly family member (3)<br>o Health worker (4)<br>o Aponjon message (5)<br>o Others___(66)	
40.	Within the first 42 days after delivery how many checkups were attended?	o 1 (1)<br>o 2(2)<br>o 3(3)<br>o More than 3 (4)	
41.	Time of first PNC	o Within __________days	Write N/A where applicable
42.	Time of second PNC	o Within __________ days	Write N/A where applicable
43.	Time of third PNC	o Within___________ days	Write N/A where applicable
44.	Time of fourth PNC	o Within __________ days	Write N/A where applicable
45.	When was the baby's first bath given?	o Right after birth (1)<br>o Within the first day (2)<br>o Within the 2nd day (3)<br>o Within the 3rd day (4)<br>o Within the 7th day (5)<br>o Others _____(66)	
46.	What was the baby fed right after birth?	o Colostrum (1)<br>o Honey (2)<br>o Sweetened water (3)<br>o Formula feeding (4)<br>o Was provided saline/oxygen due to complications (5)<br>o Others ________(66)	
47.	(If colostrum was fed) Source of knowledge on colostrum feeding	o Only Aponjon (1)<br>o Aponjon and other sources (2)<br>o Other sources (3)<br>o Don't know (98)<br>o Others___(66)	
48.	(Ask if baby is 5 or 5+ months old) What do	o Breast milk (1)<br>o Family food beside breast milk	

	you feed the baby?	(2)<br>o Others____(66)	
49.	Which food you took most after your baby was born?	Write here	
50.	Which food you avoided after the delivery of your baby?	Write here	Write N/A when appropriate
51.	Reason for avoiding this food	Write here	Write N/A when appropriate

New born and infant's immunization

	Questions	Answer	Instructions for field researcher
52.	Has the baby received any shots (vaccination)?	o Yes (1)<br>o No (2)<br>o Not applicable (99)	If No, skip to Q-55
53.	(If yes), Name the vaccines that was provided to the baby	▪ BCG<br>▪ Pentavalent<br>▪ DPT<br>▪ Polio<br>▪ Measles<br>▪ MMR<br>▪ Rota virus<br>▪ Hepatitis<br>▪ Others_____	
54.	Knowledge source behind vaccination	o Only Aponjon (1)<br>o Aponjon and other sources (2)<br>o Other sources (3)<br>o Don't know (98)<br>o Others___(66)	Skip to Q-56
55.	If vaccination was not done, what was the reason behind it?	o The baby was feverish/ caught cold<br>o Immunization centre too far from home	

		o Baby was too small, felt scared<br>o I am afraid of fever that may arise as a consequence of immunization<br>o Don't know where to go for immunization<br>o I didn't know the immunization schedule<br>o Others ______(66)	

Family planning:

	Question	Answer	Instructions for field researcher
56.	Did you uptake any family planning methods yet?	o Yes<br>o No<br>o Others___(66)	
57.	Can you tell me the birth gap that should be maintained before you conceive another baby?	o Any time/ When God permits (1)<br>o 1 year (2)<br>o 2 years (3)<br>o 3 years (4)<br>o Depends on my husband (5)<br>o I am not planning for any more kids<br>o Don't know (6)<br>o Others ___ (66)	

Access to potable drinking water

	Question	Answer	Instructions for field researchers
58.	Where do you get the pure drinking water from?	o Potable water (boiled/ safe tubewell/filter) (1)<br>o Open water(pond, river, tap etc.) (2)	If the answer is "open water", then skip to Q-60
59.	Where did you learn about potable drinking water?	o Only from Aponjon (1)<br>o Aponjon and other sources(2)<br>o Other sources (3)	

		o Don't know (98)<br>o Others_________(66)	

Hand washing practice:

	Question	Answer	Instructions for field researchers
60.	When do you wash hands everyday?<br>(multiple answers)	▪ Before saying prayers<br>▪ After waking up in the morning<br>▪ Before meal<br>▪ After meal<br>▪ After defeacation<br>▪ During bath<br>▪ Before preparing food<br>▪ While washing or cooking ingredients<br>▪ Others__	✓ Mark means (1=yes)<br>No mark (2=no)
61.	How do you wash your hands usually?	o With water (1)<br>o Water and soap (2)<br>o Water, soap , sometimes antiseptics (3)<br>o Others______(66)	
62.	Where did you learn about hand washing practice?	o Only Aponjon (1)<br>o Aponjon and other sources (2)<br>o Other sources(3)<br>o Don't know (98)<br>o Others_________(66)	

Measurement of satisfaction and feedback to the service

	Question	Answer	Instructions for field researchers
63.	Are you benefitted by Aponjon service?	o Yes (1)<br>o No (2)<br>o Others____(66)	If No, skip to Q-65
64.	If yes , Can you explain how?<br>(Multiple answers)	▪ Timely vaccination<br>▪ Take proper and ample food<br>▪ Planned for delivery<br>▪ Tested blodd group/ identified donor<br>▪ Take care of hygiene<br>▪ Call to Aponjon helpline and consult with doctor<br>▪ Received help from family	✓ Mark means (1=yes)<br>No mark (2=no)

		members<br>▪ Can take care of baby<br>▪ Learned about baby's development and growth<br>▪ Others_____	
65.	What new information did you learn from Aponjon?<br><br>(Multiple answer)	▪ Nothing<br>▪ Making eye contact with baby<br>▪ Observing baby's urination 6-7 times a day which indicates baby's development<br>▪ If baby moves and plays, it is a sign of good health<br>▪ Vaccination schedule<br>▪ Growth milestone: baby's shoulders getting strong<br>▪ How to take care of naval<br>▪ Nutrition of lactating mother<br>▪ Nutrition of baby<br>▪ Others ____(66)	
66.	Are you satisfied with Aponjon?	o Yes (1)<br>o No (2)<br>o Others ___ (66)	If yes, skip to Q-68
67.	If you are not satisfied, what is the reason behind this?<br><br>(Multiple answers)	▪ I know all the information<br>▪ Do not receive messages regularly<br>▪ Messages come at an odd time<br>▪ Service is pricy<br>▪ Could not connect to doctor at counseling line by dialing 16227<br>▪ Others____	✓ Mark means (1=yes)<br>No mark (2=no)
68.	What else do you want from Aponjon service?	Write here	
69.	Score this service between 1-5 (1= lowest performance, 5= highest performance)	o 1 (1)<br>o 2 (2)<br>o 3 (3)<br>o 4 (4)<br>o 5 (5)	

70. Special note (to be filled out as part of filed observation by researchers)
